# Neuro-cognitive foundations of word stress processing - evidence from fMRI

**DOI:** 10.1186/1744-9081-7-15

**Published:** 2011-05-16

**Authors:** Elise Klein, Ulrike Domahs, Marion Grande, Frank Domahs

**Affiliations:** 1 Department of Neurology, Section Neuropsychology, University Hospital, RWTH Aachen University, Aachen, Germany; 2 Interdisciplinary Center for Clinical Research Aachen, University Hospital, RWTH Aachen University, Aachen, Germany; 3 Institute of Psychology, Eberhard Karls University, Tuebingen, Germany; 4 Institute of Germanic Linguistics, University of Marburg, Germany; 5 Department of Neurology, Section Clinical Cognition Research, University Hospital, RWTH Aachen University, Aachen, Germany

## Abstract

**Background:**

To date, the neural correlates of phonological word stress processing are largely unknown.

**Methods:**

In the present study, we investigated the processing of word stress and vowel quality using an identity matching task with pseudowords.

**Results:**

In line with previous studies, a bilateral fronto-temporal network comprising the superior temporal gyri extending into the sulci as well as the inferior frontal gyri was observed for word stress processing. Moreover, we found differences in the superior temporal gyrus and the superior temporal sulcus, bilaterally, for the processing of different stress patterns. For vowel quality processing, our data reveal a substantial contribution of the left intraparietal cortex. All activations were modulated by task demands, yielding different patterns for same and different pairs of stimuli.

**Conclusions:**

Our results suggest that the left superior temporal gyrus represents a basic system underlying stress processing to which additional structures including the homologous cortex site are recruited with increasing difficulty.

## Introduction

It is widely agreed that the processing of spoken words comprises acoustic and phonological analysis before in a second step lexical and semantic information can be retrieved (e.g., [1-3]). With respect to the acoustic-phonological analysis of spoken words, there is general consensus that the categorical perception of phonetic properties like frequency formants, transitional properties of formants, fundamental frequency, duration, or intensity leads to the identification of strings of phonemes and - at least in languages with variable stress - to the identification of word stress patterns. On a neuro-functional level, phonological processing has been attributed to the superior temporal gyrus of both hemispheres (e.g., [4-10]). However, so far no study has aimed at directly differentiating vowel quality and word stress processing. As a starting point, findings on the processing of both vowel quality as well as stress information will be reviewed briefly.

### The autonomy of vowel quality and word stress representations

First evidence for a relative independence of vowel quality and word stress encoding in speech production came from psycholinguistic research. In particular, speech errors that involve stress exchange such as "my 'prosodic (pro'sodic) colleagues" [11], though occurring rather rarely, specifically demonstrate a separate encoding stage for word stress. Moreover, findings from speech perception point to a relatively independent processing of stress and vowel quality information although, of course, the metrical feature 'stress' inevitably has also its vowel quality correlates such as vowel reduction in unstressed syllables ([12,13]). For instance, not only minimal stress pairs (i.e., words only differing in their stress position) can be successfully discriminated on the basis of their different stress patterns; even isolated syllables excised from such minimal pairs can be reliably assigned to their source words [12,14]. Isolated syllables bearing a stressed or unstressed pitch contour can influence the processing of subsequently presented targets which have a segmentally identical initial syllable with congruent pitch [15]. However, while both vowel quality and stress can separately contribute to lexical recognition [16], there is evidence that vowel quality information can be exploited earlier than stress information (e.g., vowel duration, pitch height, and amplitude) due to coarticulation [16-19].

Findings from Dupoux and colleagues suggest that also on the level of abstract representation vowel quality and stress information may dissociate [20-22]. The so-called 'stress deafness' investigated by these authors is in fact not a difficulty to perceive and distinguish stressed and unstressed syllable patterns. Rather, only when increased memory demands come into play, participants display difficulties to remember stress patterns. More specifically, participants whose native language does not use stress to distinguish between words (e.g., French) perform significantly lower in tasks testing memory for stress patterns than participants whose language does contain minimal stress pairs (e.g., Spanish). Crucially, although French participants have particular problems in remembering stress patterns, their performance in remembering minimal pairs of pseudowords only distinguished by one consonant did not differ from the performance of Spanish participants [21]. Native speakers of German have not been tested yet, but they should obviously belong to the second class of participants, as there are minimal stress pairs like *'Tenor *vs. *Te'nor*.

Further evidence supporting the autonomy of vowel quality and word stress knowledge comes from clinical observations on brain-lesioned patients. A classical finding in aphasic word production is that there are more vowel quality errors in unstressed than in stressed syllables (e.g., [23-25]). Furthermore, a number of aphasic patients have been described showing a dissociation between spared vowel quality and impaired stress processing. Typically, their errors have been classified as regularisation related to the assignment of word stress, i.e. those patients mostly produced the regular or dominant stress pattern avoiding the irregular or infrequent pattern while preserving syllable and phoneme structures [26-33]. The reverse pattern, i.e., vowel quality errors with preserved word stress assignment is a standard finding in aphasic patients (e.g., [24]). However, there is accumulating evidence for an interaction between vowel quality and stress processing in German speech production. Data from pseudoword reading [29,34,35], EEG [36], and patient studies [31] have shown that the assignment of main stress position in German words is influenced by their vowel quality.

### Neuronal correlates underlying the processing of linguistic prosody

There is an extensive body of literature on the possible lateralization of processes involved in the comprehension of linguistic vs. emotional prosody based on neuro-imaging methods such as functional magnetic resonance imaging (fMRI). In this respect, it has been assumed that the processing of emotional prosody elicits bilateral fronto-temporal patterns (e.g. [37]), while processing of linguistic prosody has been suggested to be left lateralized in the superior temporal gyrus (for a review see [38]; but see [39] for activation of Broca's area associated with linguistic aspects of prosody). For the processing of linguistic aspects of prosody like contrastive stress and intonation, a considerable number of studies revealed a consistent involvement of the superior temporal gyrus. However, it is still under debate whether this region is involved left-lateralized or bilaterally. On the one hand, Tong et al. [40] reported significantly stronger left lateralized activation of the posterior middle temporal gyrus for the comparison of stress vs. intonation for Chinese speakers. Furthermore, Ischebeck, Friederici, & Alter [41] compared the processing of phrase boundaries in natural vs. hummed speech and identified the superior temporal gyrus extending into the sulcus to be involved bilaterally in the processing of natural speech whereas hummed speech revealed only left lateralized activation of this region. On the other hand, when Meyer, Steinhauer, Alter, Friederici, and von Cramon [42] contrasted normal speech (containing vowel quality and prosodic information) with degraded speech (lacking vowel quality information), they found bilateral activation of the posterior superior temporal gyrus even for the case of degraded speech. Taken together, previous results reported on the processing of linguistic prosody are rather heterogeneous as regards possible lateralization.

To our knowledge, up to date only one study has directly investigated the neuro-anatomical correlates of word stress processing. In an fMRI study, Aleman, Formisano, Koppenhagen, Hagoort, de Haan, & Kahn [43] asked participants to decide whether Dutch bisyllabic words were iambic (e.g., *salát*) or trochaic (e.g., *mónat*). They found areas in the left precentral gyrus, the left superior parietal lobule, and in the posterior part of the left superior temporal gyrus extending into the sulcus to be more active in this stress task compared to a semantic control condition. However, in their paradigm the identification of iambic and trochaic stress patterns relied on metalinguistic knowledge rather than on natural language processing. Such a metalinguistic task may involve more than only prosodic processing. Most importantly, contrasting a stress decision task to a semantic control condition may be not specific enough to identify regions involved in the processing of word stress (as opposed to phonological processing in general). In sum, the neural correlates underlying word stress processing are far from being understood.

## The Present Study

The current study was conducted to systematically investigate the neuronal correlates underlying word stress processing. To avoid lexical and semantic confounds on prosodic processing, we conducted an fMRI study on the processing of stress patterns using pseudowords. All stimulus items contained only stressable syllables (see [36]) which enabled us to control for vowel quality in conditions with varying stress patterns. Healthy participants were asked to state whether two auditorily presented bisyllabic pseudowords were the same or different. In the 'different' condition, items differed either in the position of word stress (e.g., *Bo'kam *vs. '*Bokam*) or in the quality of the first vowel. In the latter case, vowel quality differences were present both in stressed and unstressed syllables (e.g., '*Bekam *vs. '*Bokam *and *Be'kam *vs. *Bo'kam*). Pseudowords only contained two instead of three syllables, as we expected that the stress pattern of trisyllabic words can already be inferred after heaving heard the first two syllables [14,16,36]. Moreover, the linguistic activity of interest (i.e., the comparison of stress patterns) was contrasted with a similar phonological activity (i.e., the comparison of vowel identity) to allow the investigation of highly specific activation patterns. In contrast to previous studies (e.g., [43]), the word pairs were spoken by two different speakers: one male, one female. This way, in our stimulus-matching task we aimed at investigating the processing of stress patterns at a rather abstract (phonological) processing level not allowing for a direct comparison of phonetic values (see also [21]). Previous fMRI studies using words and pseudowords revealed that activations underlying lexical proscessing are not evoked if pseudowords are processed in a merely phonological task [44]. Given this finding, the present design should be appropriate to investigate phonological processing relatively uncontaminated by lexical or semantic search.

Building on the above considerations on the processing of phonological information the analyses were conducted in two consecutive steps. They started from examining general activation differences between different tasks addressing stress and vowel quality processing, respectively, to proceed to more specific contrasts investigating the influence of stimulus type (identical and non-identical pairs, penultimate and final stress patterns).

Note that all stimuli contained vowels and - given that they were bisyllabic - they were also marked for stress. Therefore, vowel and stress information were present in both conditions, and presumably participants automatically processed both types of information irrespective of condition. Nevertheless, the conditions differ in two crucial ways: The first difference was task instruction. In the vowel condition participants were instructed to pay attention to vowel information, whereas in the stress condition they were told to pay attention to stress information. The second difference was related to stimulus type. In the non-identical condition the two pseudo-words either differed in stress or in vowel quality. Therefore, activation observed only in the non-identical conditions may have most likely reflected stimulus-related effects, while activations seen in both identical and non-identical pairs might be related to the task manipulation (i.e. particular attention paid to stress or vowel differences).

Taken together, the main goal of the present study was to identify brain regions involved in word stress processing. Thus, we aimed at directly contrasting stress and vowel quality processing. Leaving higher linguistic processing (e.g., lexical or semantic access) aside, our study enabled us to evaluate word stress processing in more detail. Thereby, the research questions motivating the current study were twofold: (i) What is the specific activation pattern associated with word stress processing? (ii) How are activation patterns influenced by stimulus properties (same or different)? (iii) Are there any differences and/or similarities in localization and/or intensity of fMRI signal change specifically associated with the metrical processing of different stress patterns (penultimate vs. final stress)?

## Methods

### Participants

Twenty four right-handed native German-speaking healthy volunteers (12 female; mean age: 28.2 years, SD = 7.0 years) participated in this study after having given their written informed consent in accord with the protocol of the local Ethics Committee of the RWTH Aachen Medical Faculty.

### Material

A complete overview on all stimulus items used is provided in additional file 1. Stimulus material consisted of pairs of bisyllabic pseudowords obeying German phonotactic constraints. All items consisted of an initial open syllable with a single plosive in onset position followed by a closed syllable, containing simple consonantal onset and coda positions, respectively (CV.CVC). Both syllables were stressable (i.e., excluding schwa-syllables). Pairs of stimuli were created such that they either differed only with respect to word stress (stress condition) or only with respect to vowel quality (vowel condition). Furthermore, each pair consisted of one token spoken by a female and one token spoken by a male voice, respectively (see below).

**Figure 1:**
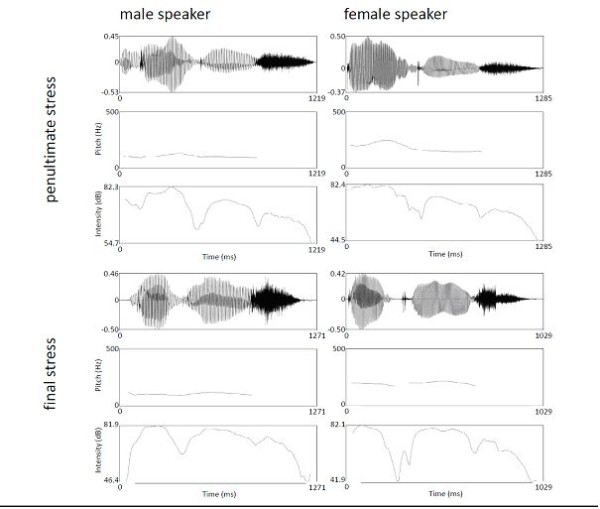
**Spectrograms, pitch contour, and intensity information for both stress patterns and speakers, illustrated with the stimulus quadruple "degis"**.

In pairs pertaining to the stress condition, two pseudowords containing the same vowels were produced with word-initial and word-final stress. Table 1 gives an overview over phonetic parameters realized by both speakers to mark stress and Figure 1 exemplifies phonetic information of the stimuli used. The examples of spectrograms, pitch, and intensity curves for both speakers and stress patterns show that phonetic prominence was clearly marked in each stress condition.

**Table 1:** Means (standard deviations are given in parentheses) of phonetic parameters duration, fundamental frequency, and intensity of the male and female speakers from a representative sample of 24 quadruples of stimuli (2 speakers × 2 stress patterns).

	Duration in seconds				Fundamental Frequency in Hz				Intensity in dB			
**stress pattern**	**PU**		**F**		**PU**		**F**		**PU**		**F**	

**syllable type**	**S1**	**S2**	**S1**	**S2**	**S1**	**S2**	**S1**	**S2**	**S1**	**S2**	**S1**	**S2**

**female speaker**	**.37 (.06)**	.73 (.07)	.30 (.06)	**.80 (.06)**	**213 (21)**	162 (28)	191 (11)	**189 (8.5)**	**80 (1.5)**	71 (4.2)	78 (2.1)	**77 (2.2)**

**male speaker**	**.47 (.05)**	.76 (.06)	.34 (.06)	**.83 (.07)**	**129 (21)**	102 (7)	119 (32)	**110 (11)**	**79 (1.1)**	73 (2.6)	77 (2.5)	**77 (1.4)**

The values for F0 and intensity are averaged over syllables using PRAAT (Boersma & Weenink; version 5.1.19). Each value is given for each syllable in each stress pattern (numbers indicate the syllable position within a word and stressed syllables are printed in bold).

PU: penultimate stress; F: final stress.

**Figure 2:**
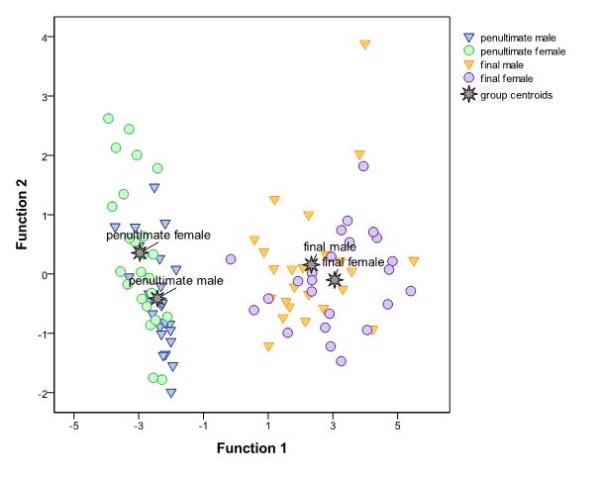
**Combined groups plot of a linear discriminant analysis on syllable-wise ratios (stressed: unstressed) of duration, fundamental frequency, and intensity for 24 representative pseudoword quadruples, revealing three discriminant functions**. Function 1 explained 98.5% of the variance, canonical R^2 ^= .89, whereas Function 2 explained only 1.1%, canonical R^2 ^= .08 and Function 3 only .4%, canonical R^2 ^= .03. In combination, all three discriminant functions significantly differentiated the conditions, Λ = .10, χ^2^(9) = 207.91, p < .001. After removing Function 1, the remaining functions still differentiated the conditions significantly, Λ = .90, χ^2^(4) = 10.17, p = .038. However, Function 3 alone did not differentiate the conditions significantly Λ = .97, χ^2^(1) = 2.58, p = .108. Note that Function 1 clearly differentiates between both stress patterns. All three phonetic variables (duration, fundamental frequency, and intensity) loaded on Function 1 (r = .93, r = -.48, and r = -.34, respectively).

As expected, there was between-speaker variance in stress realization. Consequently, Wilcoxon signed-rank tests for the syllable-wise stressed-unstressed-ratio of duration, fundamental frequency, and intensity for a representative sample of 24 pairs of tokens revealed significant between-speaker differences for fundamental frequency and intensity for the second syllable (Z ≤ -2.342; uncorrected p ≤ .017). We are aware of the problem that non-significant phonetic differences may still influence perception while the mere statistical significance of phonetic differences does not grant perceptual consequences. Nevertheless, we think that presenting tokens by a male and a female speaker should provoke a strategic shift in auditory processing, disfavoring a purely phonetic approach and encouraging a more abstract, phonological type of target comparison. Figure 2 illustrates that, indeed, phonetic means to mark word stress varied considerably both within and between speakers. At the same time it shows that stress patterns could be clearly distinguished based on a combination of three relevant phonetic variables (duration, fundamental frequency, and intensity).

In order to control for vowel quality in the stress condition, all vowels were realized as tensed. Experimental pairs contained four different vowels:/u:/,/o:/,/ø:/, and/e:/. In each pair, the difference in vowel quality invariantly affected the nucleus of the first syllable.

For the 4 vowel contrasts differing in one or two features (difference in 1 feature: between/u:/and/o:/as well as between/e/and/ø:/; in 2 features: between/ø:/and/u:/as well as between/e:/and/o:/, for an overview see Appendix) 12 item pairs as well as 12 control pairs (with identical vowels) were created. Because there was only one vowel contrast differing in 3 features (between/e:/and/u:/), 24 item pairs as well as 24 control pairs (with identical vowels) were created for this vowel contrast. This resulted in a total of 24 × 6 = 144 pseudoword pairs. Another 144 pairs of different items were used in the stress condition. These 288 pairs of different items were opposed to 288 pairs of identical items. Thus, each experimental pseudoword appeared four times in second position of a pair: (i) in the identical and (ii) in the non-identical stress condition, as well as (iii) in the identical and (iv) in the non-identical vowel condition. From this overall set blocks were determined consisting of 12 item pairs which contained each six pairs of different items (all stemming from one cell of the experimental design) and six pairs of identical items. All initial items of a given block had the same stress pattern, such that in every trial the decision could only be based on the second item of a pair.

All stimuli were spoken by two experienced native speakers of German - one female and one male - and recorded using Amadeus Pro sound editing software (Version 1.5.1, HairerSoft). In each pair presented, one item was spoken by the female and the other one by the male speaker - order being counterbalanced across conditions. Thus, strictly speaking, even 'identical pairs' were not identical on a (phonetic) 'token' level, but only on a more abstract (phonological) 'type' level of representation. This approach was chosen to increase phonetic variation and, in consequence, to highlight processes at the level of abstract phonological representations (see also [21]).

### Task and Procedure

The experiment was a combined functional magnetic resonance imaging (fMRI) and reaction time (RT) study. Participants were lying in the scanner and listening to the word pairs presented auditorily via headphones. Head movements were prevented by using soft foam pads. Participants were instructed to respond as quickly and accurately as possible avoiding unnecessary movements. To familiarize participants with the task and to reduce potential training effects during fMRI data acquisition, all volunteers were given the opportunity to practice on 16 pairs in a separate room before they entered the scanner. None of these practice items was repeated during the fMRI experiment.

The experiment was conducted in a box-car design comprising 48 blocks. Two seconds prior to the start of each block one of two specific warning sounds was presented, indicating whether the following block belonged to the stress or to the vowel condition. The assignment of warning sounds varied over participants (e.g., for half of the participants a ringing sound indicated the vowel condition and a smashing sound the word stress condition, whereas for the other half of the participants the opposite assignment was chosen). In the off-phase between blocks (duration 11.1 seconds) no audio signal was presented until the onset of the next warning sound.

Each block consisted of 12 trials (6 pairs of identical and 6 pairs of non-identical pseudowords, see Material), lasting 3700 ms per trial. Participants had to decide, whether the two items of a given pair were phonologically identical or not by pressing a button with the left (non-identical) or the right (identical) hand. The duration of the pseudowords ranged between 1000 and 1200 ms. Presentation rate of the trials was kept constant irrespective of the participants' response speed. Therefore, each block invariantly lasted 44.4 seconds. Order of trials, blocks, and speakers (male or female) was pseudo-randomized such that systematic confounds between condition (e.g. identical vs. non-identical) and stimulus order were avoided. Each participant was exposed to the same sequence of trials.

### Scanning procedure and imaging data acquisition

For each participant, a high-resolution T1-weighted anatomical scan was acquired with a 3T Siemens Magnetom TrioTim MRI system using the standard head coil (TR = 19 s, matrix = 256 × 256 mm, 190 slices, voxel size = 1 × 1 × 1 mm; FOV = 256 mm, TE = 4.9 ms; flip angle = 25°). Moreover, one functional imaging block sensitive to blood oxygenation level-dependent (BOLD) contrast was recorded for each participant (T2*-weighted echo-planar sequence, TR = 2400 ms; TE = 30 ms; flip angle = 90°; FOV = 220 mm, 88 × 88 matrix; 42 slices, voxel size = 2.5 × 2.5 × 2.5 mm, gap = 10%). Trials were presented at a rate of 3700 ms.

### Analyses

Reaction time (RT) analysis was based on correct trials only. Furthermore, response latencies faster than 200 ms were not considered and in a second step responses outside the interval of +/-3 standard deviations around the individual mean were excluded. This resulted in a total loss of 12.0% of the data. Error rates were arcsine-transformed prior to statistical analyses. RT and error rates (ER) were analyzed using a 2 × 2 × 2 within-participant repeated measures ANOVA comprising the factors identity (identical vs. non-identical pairs), phonological manipulation (stress vs. vowel condition), and stress pattern of the second item (penultimate vs. final stress).

The anatomical scans were normalized and averaged in SPM8 http://www.fil.ion.ucl.ac.uk/spm. The fMRI time series was corrected for movement and unwarped in SPM8. Images were motion corrected and realigned to each participant's first image. Data were normalized into standard stereotaxic MNI space. Images were resampled every 2.5 mm using trilinear interpolation and smoothed with a 5 mm FWHM Gaussian kernel to accommodate inter-subject variation in brain anatomy and to increase signal-to-noise ratio in the images. The data were high-pass filtered (128 s) to remove low-frequency signal drifts and corrected for autocorrelation assuming an AR(1) process. Brain activity was convolved over all experimental trials with the canonical haemodynamic response function (HRF). For activation, which was evaluated at an uncorrected p-value of < .001, cluster threshold correction was applied as a threshold larger than 12 voxels corresponded to a corrected alpha level < .05 with our parameters given. Localization of activation peaks was determined using the anatomic automatic labling tool (AAL, http://www.cyceron.fr/web/aal__anatomical_automatic_labeling.html) as well as the SPM Anatomy Toolbox [45], available with all published cytoarchitectonic maps from http://www2.fz-juelich.de/inm/index.php?index=194). Complex contrasts were masked inclusively to prevent that e.g. subtraction of a strong from a less strong deactivation suggests activation while in fact there is an underactivation.

## Results

### Behavioral data

A descriptive overview of the results is provided in Table 2. The ANOVA of RT data revealed main effects of identity and phonological manipulation [F(1, 23) ≥ 39.50, p ≤ .001], indicating that decisions were faster on non-identical pairs than on identical pairs (1100 ms vs. 1173 ms) and faster for vowel contrasts than for stress contrasts (1081 ms vs. 1192 ms). There was no main effect of stress pattern [F(1, 23) = 2.77, p = .109]. Moreover, there was a significant interaction of identity and phonological manipulation [F(1, 23) ≥ 26.45, p ≤ .001], meaning that the disadvantage for identical as compared to non-identical pairs was more pronounced in the vowel condition (1140 ms vs. 1021 ms) than in the stress condition (1206 ms vs. 1179 ms). None of the other two- or three-way interactions reached statistical significance.

**Table 2:** Overview of behavioral results.

		identical		non-identical	
		
		Penultimate	Final	Penultimate	Final
**phonological manipulation**	**stress**	1215.7 (203.9) 9.5 (4.4)	1195.5 (204.5) 8.3 (5.8)	1177.9 (218.2) 9.5 (5.4)	1179.3 (206.1) 17.6 (6.7)
	
	**vowel**	1147.9 (237.6) 5.8 (5.5)	1131.8 (220.6) 13.1 (5.3)	1022.5 (220.0) 9.4 (5.3)	1020.2 (216.5) 13.8 (6.1)

Mean RT (SD) in ms given in the first line and mean error rates (SD) in % given in the second line of each cell.

The ANOVA of arcsine-transformed error-rates yielded significant main effects of both identity and stress pattern [F(1, 23) ≥ 7.52, p ≤ .012], while the main effect of phonological manipulation only approached the conventional level of significance [F(1, 23) ≥ 3.12, p = .091]. Specifically, non-identical trials were somewhat more error prone than identical trials (12.6% vs. 9.2%), second items with final stress led to more errors than second items with penultimate stress (13.2% vs. 8.6%), and decisions in the stress condition tended to be less accurate than those in the vowel condition (11.2% vs. 10.5%). While there was no significant three-way interaction, all two-way interactions were significant or marginally significant [F(1, 23) ≥ 3.77, p ≤ .064]. Specifically, the increase in error rates from identical to non-identical was particularly pronounced for second items with final compared to penultimate stress (final stress: 10.7% to 15.7%, penultimate stress: 7.7% to 9.4%) and for the stress condition compared to the vowel condition (stress condition: 8.9% to 13.5%, vowel condition: 9.5% to 11.6%). The advantage for penultimate compared to final stress was more pronounced in the vowel condition than in the stress condition (vowel condition: 7.6% to 13.4%, stress condition: 9.5% to 12.9%).

### fMRI data

Analysis of fMRI data was based on all trials. In a first step, a conjunction over the contrasts of stress vs. baseline and vowel quality vs. baseline was conducted to show the largely overlapping cortical areas, which were activated in both contrasts. The baseline covered the rest periods between the blocks, in which no stimulus material was presented.

#### Conjunction over the contrasts stress vs. baseline and vowel quality vs. baseline (see Figure 3, Table 3)

**Figure 3:**
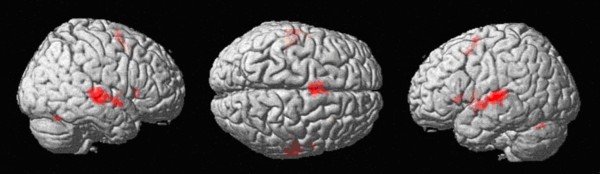
**Conjunction over the contrasts stress - baseline and vowel quality - baseline: **widespread activation in the bilateral superior temporal cortices (FWE-corrected voxelwise p < .05, cluster size k = 12).

**Table 3:** Comparison of stress- and vowel quality-related activation.

Contrast	Brain region (BA)	TC (x, y, z)			Cluster size	z score
Conjunction*	LH superior temporal gyrus (BA22)	-48	-20	8	264	6.61
Stress - baseline and	RH superior temporal gyrus (BA 22)	63	-25	0	236	6.54
Vowel quality vs. Baseline	RH insula	30	23	5	20	5.87
	LH insula	-33	20	3	27	5.84
	LH putamen	-25	3	-3	60	5.71
	RH putamen	25	3	5	17	5.38
	LH supplementary motor area (BA 6)	0	0	65	114	6.14
	RH cerebellum	30	-65	-25	24	6.17
	LH cerebellum	-28	-68	-25	21	6.04
						
Stress - vowel quality	LH inferior frontal gyrus (BA 47)	-48	18	-8	17	4.36
	RH inferior frontal gyrus (BA 44)	53	15	20	36	4.33
	RH inferior frontal gyrus (BA 45)	50	23	-5	15	4.06
	RH superior temporal gyrus (BA 22)	58	-35	18	14	3.73
	RH middle temporal gyrus (BA 21)	50	-23	-5	25	3.80
	RH superior frontal gyrus (BA 6)	8	0	68	39	4.89
						
Non-identical pairs:	RH middle temporal gyrus (BA 21)	55	-43	8	93	5.41
Stress - vowel quality	LH inferior temporal gyrus (BA 21)	-43	-3	-20	23	4.47
	LH inferior frontal gyrus (BA 47)	-48	18	-8	36	4.66
	RH inferior frontal gyrus (BA 45)	53	23	-5	43	4.52
	RH inferior frontal gyrus (BA 44)	50	13	20	60	4.02
	RH superior temporal gyrus (BA 22)	55	-25	-3	26	3.81
	LH superior temporal gyrus (BA 22)	-50	-40	20	25	3.71
	LH insula	-33	20	8	34	4.32
	RH intraparietal sulcus (BA 7)	43	-45	60	18	3.91
	RH intraparietal sulcus (BA 7)	43	-40	50	14	3.64
	RH postcentral gyrus (BA 1)	48	-23	50	54	4.10
	RH superior frontal gyrus (BA 6)	8	3	68	50	5.34
	LH middle frontal gyrus (BA 6)	-40	0	55	14	3.95
	RH middle frontal gyrus (BA 8)	45	8	40	19	3.71
	LH cingulate gyrus (BA 32)	-5	18	40	87	5.07
						
Identical pairs:	LH intraparietal sulcus (BA 7)	-35	-65	43	16	3.74
Vowel quality - stress						

*p < .05, FWE-corrected; p < .001, uncorrected; cluster size = 12 voxels; masks were created at uncorrected p < .05; MNI: Montreal Neurological Institute coordinates.

The conjunction revealed large common clusters of activated voxels, bilaterally, in the superior temporal gyri (BA 22), the insula, the putamen as well as the cerebellum (*p *< .05, FWE-corrected, *k *= 12 voxels).

In a second step, contrasts between vowel quality and stress processing were calculated to evaluate the regions found to be specifically active in word stress processing by Aleman et al. [43].

#### Stress vs. vowel quality (see Figure 4A, Table 3)

**Figure 4:**
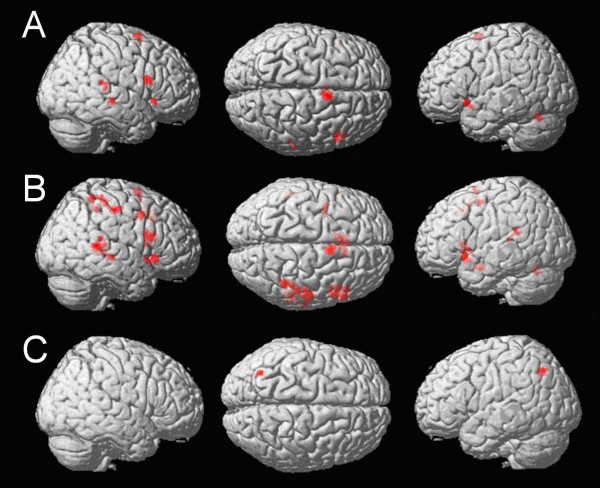
**Comparisons of stress and vowel quality. **A: Stress - vowel quality at an uncorrected voxelwise p < .001 and cluster size k = 12 voxels, masked inclusively with stress: Activation specific for prosodic processing in the right superior temporal gyri as well as in Broca's area. B: Stress vs. vowel quality in non-identical pairs at an uncorrected voxelwise p < .001 and cluster size k = 12 voxels, masked inclusively with stress in non-identical pairs reveals a widespread right-lateralized temporo-parieto-frontal network. C: Vowel quality vs. stress in identical pairs at an uncorrected voxelwise p < .001 and cluster size k = 12 voxels, masked inclusively vowel quality in identical pairs: Activation of the left intraparietal cortex.

Word stress was contrasted to vowel quality at an uncorrected voxelwise *p *< .001 and a cluster size of 12 voxels. This comparison indicated activation in the left inferior frontal gyrus [Brodmann Area (BA) 47], the right superior temporal gyrus (STG, BA 22), the right inferior frontal gyrus (BA 44, BA 45), the right middle temporal gyrus (BA 21), the left fusiform gyrus (BA 19), and the right supplementary motor area (BA 6). Please note that the activation in the STG is not lateralized on the right hemisphere for the main effect of stress vs. vowel quality. Flipping another sample of the contrast images along the y-axis (from left to right orientation) and calculating a paired t-test between the left STG in the original data set and the left STG in the flipped data set (formerly the right STG) revealed that even at a very liberal p-value < .05 there was no significant difference of activation in the STG between the two hemispheres.

More in-depth examination of this main effect revealed that the activation observed was mainly driven by differences between stress and vowel quality in non-identical trials: Whereas in trials with identical pairs the comparison of stress with vowel quality revealed no activated voxels at the threshold chosen (*p *< .001, uncorrected, *k *= 12 voxels), trials with non-identical pairs yielded a large network of activation when comparing stress to vowel quality:

#### Stress vs. vowel quality in non-identical pairs (Figure 4B, Table 3)

Contrasting stress and vowel quality in non-identical word pairs (*p *< .001, uncorrected, *k *= 12 voxels) revealed activation in the bilateral superior temporal gyrus (BA 22), the bilateral middle temporal gyrus (BA 21), the left inferior frontal gyrus (BA 45), and the right inferior frontal gyrus (BA 45 and 44). Further clusters of activated voxels were found in the left insula, the right intraparietal sulcus (BA 7), the right superior parietal lobule (BA 7), the right postcentral gyrus (BA 2), the right supplementary motor area (BA 6), the left precentral gyrus (BA 6), the right middle frontal gyri (BA 8), and the left superior medial gyrus (BA 32).

Inspection of the inverse contrast (vowel quality vs. stress) revealed no clusters of activated voxels at the threshold chosen (*p *< .001, uncorrected, *k *= 12 voxels). However, closer inspection of the data indicated activation when identical pairs were presented. In contrast, there was no activation observed for non-identical pairs.

#### Vowel quality vs. stress in identical pairs

In identical pairs, vowel quality was contrasted with stress at an uncorrected voxelwise *p *< .001 and cluster size of 12 voxels (Figure 4C, Table 3). Activated voxels were observed in the left intraparietal sulcus (BA 7).

Taken together, there was a temporo-frontal activation pattern specifically associated with word stress processing. Neural correlates underlying vowel quality processing could best be identified comparing identical pairs. An increase of the fMRI signal with vowel quality processing in the difficult condition was found in the left intraparietal cortex.

In a second step, we aimed at comparing activation patterns for targets with different stress patterns.

#### Penultimate vs. final stress in the stress condition (Figure 5A, Table 4)

**Figure 5:**
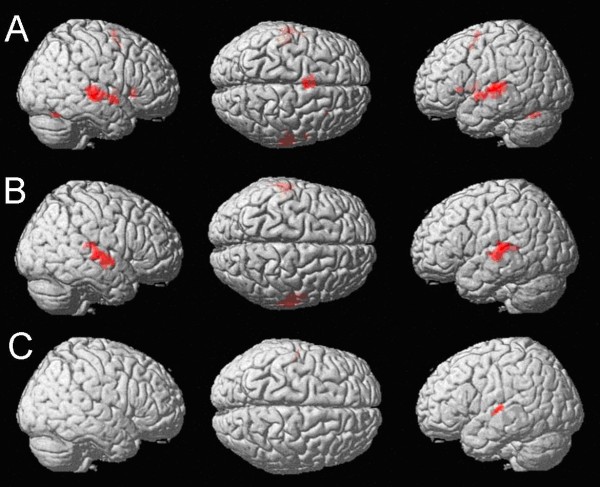
**Comparisons of penultimate and final stress.** A: Main effect of penultimate vs. final stress (FWE-corrected, cluster size *k *= 12 voxels, masked inclusively): Activation of a bilateral temporo-frontal network. B: Penultimate vs. final stress when comparing identical pairs according their stress at an uncorrected voxelwise p < .001 and cluster size k = 12 voxels, masked inclusively: Activation of the bilateral superior temporal gyri. C: Penultimate vs. final stress when comparing non-identical pairs regarding their stress at an uncorrected voxelwise p < .001 and cluster size k = 12 voxels, masked inclusively: Left-lateralized activation of the superior temporal gyrus.

**Table 4:** Penultimate and final stress: Main effect and contrasts depending on the identity of the word pair presented.

Contrast	Brain region (BA)	MNI (x, y, z)			Cluster size	z value
Stress pattern:	LH superior temporal gyrus (BA 22)	-48	-20	8	261	6.49
Penultimate - final stress*	RH superior temporal gyrus (BA 22)	63	-25	0	231	6.36
	LH putamen	-23	5	3	72	6.13
	RH putamen	25	0	5	20	5.67
	RH insula lobe	30	25	3	33	5.79
	LH insula lobe	-30	20	3	23	5.66
	LH supplementary motor area (BA 6)	-3	0	65	86	6.31
	RH cerebellum	28	-63	-25	32	6.55
	LH cerebellum	-28	-68	-25	50	6.02
	LH cerebellum	3	-63	-25	18	5.87
						
Stress in identical pairs:	LH superior temporal gyrus (BA 22)	-55	-25	8	192	4.73
Penultimate - final	RH superior temporal gyrus (BA 22)	65	-20	3	208	5.80
						
Stress in non-identical pairs:	LH superior temporal gyrus (BA 22)	-50	-18	5	23	4.10
Penultimate - final						

* p < .05, FWE-corrected; p < .001, uncorrected; cluster size = 12 voxels; masks were created at uncorrected p < .05; MNI: Montreal Neurological Institute coordinates.

Stronger activation was found in a large bilateral temporo-frontal network (FWE-corrected at *p *< .05, *k *= 12 voxels). The network comprised the bilateral superior frontal gyri (BA 22), the bilateral putamen, the bilateral insula, the left supplementary motor area (BA 6) as well as the bilateral cerebellum.

#### Penultimate vs. final stress when comparing identical pairs in the stress condition (Figure 5B, Table 4)

Activation specific for penultimate stress in identical pairs was present in large clusters in both superior temporal gyri (BA 22) extending along the superior temporal sulcus (uncorrected *p *< .001, *k *= 12 voxels).

#### Penultimate vs. final stress when comparing non-identical pairs in the stress condition (Figure 5C, Table 4)

Comparing conditions with penultimate stress to conditions with final stress in non-identical word pairs, only activation in the left superior temporal gyrus was observed (BA 22) at an uncorrected *p *< .001 and voxel size of *k *= 12 voxels.

For the opposite comparison (final vs. penultimate stress) no activation was observed at the threshold chosen neither for identical nor for non-identical pairs.

Taken together, in the superior temporal gyrus as well as the superior temporal sulcus differential effects of stress processing were found dependent on both the factors identity (non-identical vs. identical auditory word pairs) and stress pattern (penultimate stress vs. final stress).

## Discussion

The current study aimed at investigating the processing of word stress information. For this purpose, behavioral and neuro-imaging data of word stress and vowel quality processing were contrasted directly. In general, the neural networks associated with word stress and vowel quality processing were observed to be largely overlapping. In particular, the conjunction of stress and vowel tasks revealed that both aspects of phonological processing involved a network of bilateral fronto-temporal activation, resembling patterns previously described to subserve auditory processing of bisyllabic pseudo-words [46]. However, while the general contrast between stress and vowel conditions showed only right-hemispheric activation of the superior temporal gyrus, the more fine-grained analysis over non-identical pseudoword pairs showed that a bilateral fronto-temporal network was specifically associated with word stress processing. In particular, we were able to identify task-specific differences of stress processing in the superior temporal gyrus and the superior temporal sulcus. Finally, our data suggested higher cognitive demands for the processing of penultimate stress compared to final stress in the experimental design chosen.

### Stress vs. vowel quality processing

Main purpose of the present study was to evaluate the neural correlates of phonological word stress processing by comparing these correlates to those related to a similar task - vowel quality processing. The difference of stress and vowel quality processing in general was corroborated by the effect of phonological manipulation which was significant in the ANOVA on RT and marginally significant in the ANOVA on ER, meaning that reaction times were faster for vowel contrasts than for stress contrasts. In line with previous studies investigating activation related to prosodic sentence processing, the comparison of stress and vowel quality processing revealed a network of activation comprising the right superior temporal gyrus. This brain region has been identified repeatedly to be associated with prosodic processing (e.g., [38,40-42]). Moreover, activation was evidenced in Broca's area, which has also been found to be associated with linguistic aspects of prosody [39]. Finally, increased occipital activation extending into the left fusiform gyrus was observed, where the visual identification area for word forms is supposed to be located [47]. This activation may indicate that participants also searched for associations with familiar word forms and their stress patterns whenever they had to process stress information in pseudowords (see also [48]).

Comparing vowel quality and stress processing revealed no super-threshold activation in the whole brain. However, as the behavioral analysis revealed a strong impact of the factor identity, a more fine-grained analysis which takes this factor into account seems to be more adequate. Indeed, breaking down the task-specific interaction between stress/vowel quality processing and the factor identity into its constituting conditions revealed that the activation observed in the comparison of stress to vowel quality processing was mainly driven by trials with non-identical pairs. In such a comparison, not only the same areas were observed which were found to be active in the main effect of stress vs. vowel quality processing, but also the superior temporal gyrus was activated bilaterally. In addition, the right intraparietal cortex was activated. This cortex site has been suggested to underlie the processing of proximity relations [49] as well as mental imagery (e.g., [50]). Thus, the non-identical stress patterns may have been evaluated with respect to the relation and extent of their differences; moreover, participants may have tried to internally memorize and compare the stress patterns they had been presented with. It should be pointed out, that stress is an inherently relational property, i.e., its recognition requires the comparison of phonetic measures (e.g., duration, pitch, and intensity) between stressed and unstressed syllables and this relation may even be different within and between different speakers as in our task (see Figures 1 and 2 and Table 1).

In contrast, for identical pairs no activation was observed for stress vs. vowel quality processing. However, the opposite contrast showed that within identical pairs, vowel quality compared to stress processing was related to stronger intraparietal activation in the left hemisphere. This is in line with previous findings comparing vowel quality (flattened without prosody) and natural speech [42] as these data already suggested that the left intraparietal cortex may be associated with vowel quality processing.

To sum up, our imaging data indicate different activation patterns for vowel quality and stress processing when contrasting these two aspects of phonological processing directly in different stimulus context (identical/non-identical pairs). For stress activation in the context of non-identical pairs, a widespread pattern of temporo-frontal activation was observed, while the processing of vowel quality information vs. stress processing in the context of identical pseudoword pairs seems to be associated with the intraparietal cortex as already reported by Meyer et al. [42]. However, as already suggested by the behavioral data, it is important to note that both the effect of word stress and the effect of vowel quality information have to be evaluated in the context of the stimulus type (identical or different). The contrasts between vowel quality and stress processing seem to reflect qualitative differences rather than being only related to different degrees of difficulty.

### Stimulus specific effects on prosodic processing

The present study revealed that the type of stimulus pair (identical vs. different) influenced stress processing. The effect of identity was significant in the ANOVA on both RT and ER, with identical pairs being classified more slowly than non-identical pairs. Moreover, the present neuro-imaging data clearly indicated the importance of stimulus-specific effects for the above described network of activation for stress processing: Whenever a pair of pseudowords with non-identical stress patterns had to be decided on, a large bilateral network in the superior temporal gyrus was activated, which has repeatedly been identified to be vitally involved in processing prosodic information (e.g., [38,41]). However, when the stress pattern in the pair of pseudowords was identical, no activation was observed.

Mean RTs in the present study were faster for non-identical than for identical stimulus pairs, whereas in some behavioral experiments reported in the literature involving same-different decisions on vowel-consonant syllables, faster mean RTs were obtained for the processing of same syllables compared to different ones (e.g., [51,52]). However, the difference between "same" and "different" responses is subject to specific task demands (e.g., [51-53]. In the present investigation even in the "same" condition items were actually not identical but realized by different speakers. Listeners therefore could not rely on superficial phonetic deviations in the "different" condition but had to derive abstract representations to perform the evaluation task. Since the phonetic deviations in the "same" condition were more fine-grained compared to the "different" condition, the latencies for "same"-decisions were higher. However, the asymmetrical neurophysiological effect of the matching task on stress vs. vowel processing indicates qualitatively different demands on positive or negative responses.

The finding of stress processing being influenced by stimulus specific effects is relevant regarding the possible lateralization of processes which subserve the comprehension of linguistic prosody. As already outlined above, a consistent involvement of the superior temporal gyrus has been shown frequently for the processing of linguistic aspects of sentence prosody like contrastive stress and intonation [40]. However, it still remains debatable whether this region is involved only in the left hemisphere or rather bilaterally. On the one hand, a considerable number of studies reported significantly stronger left lateralized activation of the posterior middle temporal gyrus for processing stress information (e.g., [38,40]). On the other hand, bilateral activation of the posterior superior temporal gyrus has also been reported repeatedly for processing prosodic information in natural (e.g., [41]) and degraded [42] speech.

The current study may add to the understanding of such apparently heterogeneous findings. When only comparing main effects such as the main effect of stress to the main effect of vowel quality processing, only lateralized activation of the superior temporal gyrus was found. However, as outlined above, our behavioral data indicated that the identity or non-identity of stress patterns may be relevant. Indeed, when the processing of stress information was evaluated in the context of the stimulus type (identical or non-identical), bilateral activation of the superior temporal gyrus was found, which seems to correspond well to the findings of Ischebeck et al. [41] as well as Meyer and colleagues [42]. In contrast, the processing of identical stress patterns as well as a comparable contrast in the vowel task within non-identical items did not reveal such an activation pattern.

Taken together, diverging previous results regarding the lateralization of prosodic processing may have possibly been due to stimulus- or task-specific properties (see also [54] for task specific effects on neural activation patterns in two language groups requiring different efforts in the processing of stress properties). Taking these properties into account, our data suggest that whenever more fine-grained decisions have to be made at an increasingly abstract level, bilateral activation of the superior temporal gyri is needed. This view fits well with previous observations on bilateral processing of stress comparison [41,42].

### Effects of stress patterns

The present study also revealed different behavioural and imaging results for different stress patterns. The effect of stress pattern was significant in the ANOVA on error rates, with final stress in the second item being more difficult to be processed than penultimate stress. Stimulus-specific effects again influenced performance as the increase in error rates was particularly more pronounced for pairs with non-identical stress patterns.

Regarding the main effect of stress patterns, our fMRI data yielded different results than the behavioral data. In particular, no activation was found for final stress as compared to penultimate stress. However, the inverse contrast revealed a bihemispheric activation of the superior temporal gyrus, which has been repeatedly reported to be associated with prosodic processing (e.g., [40,41]). This finding suggests that the processing of penultimate stress may have involved a more detailed auditory analysis than the processing of final stress. On a phonetic level of explanation, this may have been due to the different perceptual saliency of both patterns. On a phonological level, this activation pattern may indicate that penultimate stress has not a general default status in German as already argued by Janßen, Domahs, and colleagues [29,31,36]. This is a challenge to approaches assuming that given the fact that penultimate stress (or in bisyllabic words: initial stress) is the most frequent German stress pattern it forms some kind of default stress pattern which- in contrast to final stress - has not to be lexically specified (e.g., [55,56]). However, Janßen [29], Janßen & Domahs [31] and Domahs et al. [36] report behavioral and electrophysiological evidence that the "regularity" of word stress is strongly influenced by the structure of the final and penultimate syllable [30,36,57,58]. In particular, penultimate stress occurs predominantly in words with an open final syllable (e.g., *Pánda*, [panda]), but not in words with a closed final syllable (e.g. *Spinát*, [spinach]), casting doubts on a structure-independent default status of penultimate stress. Since the pseudowords presented consist of an open penultimate and a closed final syllable, the higher processing costs for items with penultimate stress may reflect the fact that this pattern is not preferred in words with a closed final syllable (see also [34]). Again, a more fine-grained analysis of the imaging data revealed that the factor identity differentially influenced the results. Activation observed for identical stress patterns was found bilaterally, whereas the processing of non-identical stress patterns was only associated with left-lateralized activation. This distribution of activation may be explained by the following arguments. Most probably, it may have been easier to decide that two stress patterns are different than to decide that two tokens of the same stress pattern, produced by different speakers, are indeed identical at a phonological level. This assumption is supported by our behavioural data showing that responses for non-identical pairs were significantly faster than for identical pairs. However, it is important to consider that the difference in the neuro-functional data is restricted to the superior temporal gyrus, while it does not seem to involve areas associated with generally higher levels of working memory or attentional load (e.g., the dorsolateral prefrontal cortex and/or the intraparietal cortex) where activation would be expected if the different performance on identical and non-identical stimuli is purely ascribed to higher memory load. Thus, the greater activation for identical pairs seems to be rather specific to the processing of stress information itself than to reflect more general processes associated with a higher level of working memory and/or attentional demands. Thereby, the increased activation may reflect most likely the extended auditory evaluation of the more fine-grained phonetic differences in pairs with identical stress patterns.

Taken together, diverging previous results regarding the lateralization of prosodic evaluation may have possibly been due to stimulus- or task-specific properties. Taking these properties into account, our data support the view that the left posterior superior temporal gyrus is a kind of basic system mainly involved in the evaluation of prosodic properties as outlined in part of the previous literature (e.g., [40]). However, once more fine-grained decisions have to be made at an increasingly abstract level, the right superior temporal gyrus seems to be called for assistance (e.g., [54]). This view fits well with previous observations of bilateral processing related to rather abstract stress comparison, e.g., in degraded speech [42]. Thus, the present finding again underlines the impact of task and stimulus-specific effects.

### Evaluation and perspectives

We believe that the current study is a first step towards a more comprehensive understanding of the underlying processes subserving word stress processing. However, there are still a lot more steps to go. Therefore, in the remainder of this Discussion some points requiring further investigation will be addressed.

Consider first that the responses to word stress evaluation were significantly slower and tended to be more error prone than the evaluation of vowel quality information. The question may arise whether the stress condition was generally more difficult than the vowel quality condition - a methodological artefact potentially fateful for the validity of our data and the conclusions we have drawn.

However, support for the validity of our data comes from several different aspects. First, the above mentioned RT-findings neglect that the pattern observed is driven by a speed-accuracy trade-off as the slower condition also tended to be less error prone. Second, inspection of the imaging data provides helpful insights. Indeed, the comparison of stress with vowel quality processing revealed a bilateral network of activation, whereas the contrast of vowel quality vs. stress showed no voxel in the whole brain activated significantly stronger at the threshold used. However, a more fine-grained analysis showed that the activation observed for stress vs. vowel quality processing was in fact driven by the comparison of stress and vowel quality processing in trials with non-identical pairs, while for identical pairs no activation was observed for stress vs. vowel quality processing. On the contrary, within identical pairs stronger activation was observed for vowel quality as compared to stress processing. To sum up, our imaging data indicate that both the effect of word stress as well as the effect of vowel quality information have to be evaluated in the context of the stimulus type (identical or non-identical), as was already suggested by the behavioral effect of stimulus type (identical or non-identical word pairs). Thereby, the differences between vowel quality and stress processing seem to be qualitative rather than only being related to different degrees of difficulty. This interpretation is further supported by the facts that areas typically associated with higher cognitive demands (e.g., left dorsolateral prefrontal gyrus, anterior cingulate or intraparietal cortices) were not observed for the comparison of stress and vowel quality processing. Quite the contrary, in the identity condition, the intraparietal cortex was in fact significantly stronger involved in the processing of vowel quality than of stress information. This finding leads us to the question why response latencies were generally longer when evaluating stress patterns. In the literature, there is evidence that vowel quality information can be exploited earlier than stress information due to coarticulation [16-19]. Nevertheless, in order to explain the extent of these differences, it may be helpful to consider that our design enabled participants to decide on the vowel quality structure as soon as the first syllable of the second item was encountered. In contrast, for decisions on stress information, the second syllable of the second item had to be perceived before a confident judgement was possible. This explanation may account for a general difference of 100-200 ms in response latencies. Indeed, inspection of the behavioral data revealed that all stress conditions were evaluated systematically slower than the vowel quality conditions (see Table 2). Taking all these arguments into account, the current paradigm seems to be a valid approach to further investigate the neural correlates of processing word stress and vowel quality information.

Consider next the effect of stress patterns. The comparison between stress patterns revealed a bihemispheric activation of the superior temporal gyrus for penultimate stress compared to final stress. This finding suggests that the processing of penultimate stress produced higher costs than the processing of final stress. At which level of processing may pseudowords with penultimate stress have been harder to process than pseudowords with final stress? The activation differences in the identity condition may just reflect higher efforts at the level of phonetic analysis. Unfortunately, knowledge about the perceptual consequences of specific phonetic features in word stress processing is still lacking. In consequence, a perceptual account of the activation differences cannot be excluded with the data at hand. At a more abstract phonological level of processing it may be speculated that penultimate stress is generally more difficult to be processed or represented than final stress. However, such an interpretation would not be warranted as penultimate stress is the statistically predominant pattern in German words and, thus, is not likely to evoke higher costs in processing than the less frequent pattern. However, note that all stimuli presented contained a heavy final syllable and therefore do not fit the typical pattern of German words with penultimate stress, namely bisyllabic words with a light or reduced final syllable. Thus, the higher processing costs may support quantity sensitive approaches on German stress assignment [30,36,57,58] and show that for words with a heavy final syllable penultimate stress is not the unmarked pattern (see also [34]). Further neuro-functional examinations with varying syllable structures should bring more light into this debate.

Even if we assume that a difference in phonetic parameters may have affected our results, it is important to note that this is clearly not the case for our behavioral data. First, there was no main effect of stress pattern in the ANOVA on RT. The analysis of error rates even provided evidence in favour of the assumption that final stress may have been more difficult to be processed than penultimate stress as final stress in the second word of a pair was associated with significantly more errors than penultimate stress. Taking into account the phonetic parameters we do not claim that the differential imaging effects found for specific stress patterns (penultimate vs. final stress) in our study can be generalized to studies using other stimuli, presentation formats or tasks. However, the present study definitely shows that these specific stress patterns may be processed differently and should be target of further investigations, for instance, with more precisely controlled phonetic parameters and different syllable structures. Imaging studies on different stress patterns may be a crucial source of evidence feeding phonological theories on stress systems.

Taken together, even though there are still a number of questions to be answered, the present results provide first evidence not only on the neural correlates subserving stress processing, but also for the impact of stimulus-dependent effects (e.g., whether the stress/vowel quality decision has to be made within identical or non-identical stimuli).

## Summary and Conclusion

The current study addressed two main research issues: First, we were interested in the activation pattern associated with stress processing. By controlling stimulus material for vowel quality in conditions with varying stress patterns and by varying phonetic realizations we intended to provoke a matching of stress patterns on a rather abstract, phonological level.

We observed a fronto-temporal network basically comprising the right superior temporal gyrus extending into the sulci as well as the inferior frontal gyri, bilaterally, to be specifically associated with stress processing. However, when the contrast was evaluated more specifically in the context of the stimulus type (identical/non-identical pairs), the data became clearer and revealed that stress was processed in the bilateral superior temporal gyri and sulci in the more difficult non-identical trials. For vowel quality processing, our data emphasize a substantial contribution of the left intraparietal cortex.

Second, our data suggest that higher cognitive demands were needed for processing penultimate compared to final stress possibly suggesting that penultimate stress has not a default status in German. Thereby, our results support the view that the left superior temporal gyrus represents a kind of basic system underlying stress processing to which additional structures including the homologous cortex site are recruited with increasing difficulty.

## Competing interests

The authors declare that they have no competing interests.

## Authors' contributions

FD, UD, EK, and MG conceived the study. All authors participated in its design. EK performed data collection, processing and statistical analyses. EK, FD, and UD drafted the manuscript. All authors contributed to the interpretation of the data. All authors read and approved the final manuscript.

## Supplementary Material

Additional file 1 **Lists of pseudowords used**. Click here for file
