# Supplementary material for: Neuro-cognitive foundations of word stress processing - evidence from fMRI
Source: Behav Brain Funct. 2011 May 16;7:15. doi: 10.1186/1744-9081-7-15 (PMC3120660; doi:10.1186/1744-9081-7-15)
Supplement: Additional file 1 — Lists of pseudowords used. [file 1744-9081-7-15-S1.DOC]

**Additional material:** Lists of pseudowords used.

|  | **Vowel quality** | | **Experimental pairs** | | | |
| --- | --- | --- | --- | --- | --- | --- |
|  | **Feature** | | **Penultimate stress** | | **Final stress** | |
|  | **difference** | | **Vowel quality** | **Stress** | **Vowel quality** | **Stress** |
| 1 | 1 | /o:/-/u:/ | gúdam - gódam | gútum - gútum | b**ó**sil - b**ó**sil | gódam - godám |
| 2 | 1 | /o:/-/u:/ | túbon - tóbon | púgus - púgus | d**ó**suf - d**ó**s**u**f | tóbon - tobón |
| 3 | 1 | /o:/-/u:/ | p**ú**fat - p**ó**fat | dólan - dólan | g**ó**bal - g**ó**bal | p**ó**fat - pof**á**t |
| 4 | 1 | /o:/-/u:/ | bútul - bótul | túfam - túfam | k**ó**lop - k**ó**lop | bótul - botúl |
| 5 | 1 | /o:/-/u:/ | d**ú**tap - d**ó**tap | kúbuf - kúbuf | p**ó**tan - p**ó**t**a**n | d**ó**tap -dot**á**p |
| 6 | 1 | /o:/-/u:/ | kútam - kótam | búraf - búraf | t**ó**fam - t**ó**fam | kótam - kotám |
| 7 | 1 | /e:/-/ø:/ | pöwal - péwal | töbon - töbon | b**é**tul - b**é**tul | péwal - pewál |
| 8 | 1 | /e:/-/ø:/ | tökap - tékap | g**ö**tum - g**ö**tum | d**é**rom - d**é**rom | tékap - tekáp |
| 9 | 1 | /e:/-/ø:/ | köbut - kébut | pötan - pötan | g**é**san - g**é**san | kébut - kebút |
| 10 | 1 | /e:/-/ø:/ | bösil - bésil | d**ö**gis - d**ö**gis | k**é**lop - k**é**lop | bésil - besíl |
| 11 | 1 | /e:/-/ø:/ | dömil - démil | kötam - kötam | p**é**gus - p**é**gus | démil - demíl |
| 12 | 1 | /e:/-/ø:/ | göbal - gébal | bögom - bögom | t**é**fam - t**é**fam | gébal - gebál |
| 13 | 1 | /u:/-/o:/ | gótum - gútum | gódam - gódam | b**ú**gom - b**ú**gom | gútum - gutúm |
| 14 | 1 | /u:/-/o:/ | pógus - púgus | tóbon - tóbon | d**ú**wal - d**ú**wal | púgus - pugús |
| 15 | 1 | /u:/-/o:/ | dólan - dúlan | p**ó**fat - p**ó**fat | g**ú**sif - g**ú**sif | dúlan - dulán |
| 16 | 1 | /u:/-/o:/ | tófam - túfam | bótul - bótul | k**ú**fat - k**ú**fat | túfam - tufám |
| 17 | 1 | /u:/-/o:/ | kóbuf - kúbuf | d**ó**tap - d**ó**tap | p**ú**sim - p**ú**sim | kúbuf - kubúf |
| 18 | 1 | /u:/-/o:/ | bóraf - búraf | kótam - kótam | t**ú**kap - t**ú**kap | búraf - buráf |
| 19 | 1 | /ø:/-/e:/ | tébon - töbon | péwal - péwal | b**ö**raf - b**ö**raf | töbon - töbón |
| 20 | 1 | /ø:/-/e:/ | g**é**tum - g**ö**tum | tékap - tékap | d**ö**tap - d**ö**tap | g**ö**tum - göt**ú**m |
| 21 | 1 | /ø:/-/e:/ | pétan - pötan | kébut - kébut | g**ö**sif - g**ö**sif | pötan - pötán |
| 22 | 1 | /ø:/-/e:/ | d**é**gis - d**ö**gis | bésil - bésil | k**ö**buf - k**ö**buf | d**ö**gis - dög**í**s |
| 23 | 1 | /ø:/-/e:/ | kétam - kötam | démil - démil | p**ö**sim - p**ö**sim | kötam - kötám |
| 24 | 1 | /ø:/-/e:/ | bégom - bögom | gébal - gébal | t**ö**bar - t**ö**bar | bögom - bögóm |
| 25 | 1 | /o:/-/u:/ | godám - godám | bos**í**l - b**ó**sil | bus**í**l - bos**í**l | bug**ó**m - bug**ó**m |
| 26 | 1 | /o:/-/u:/ | tobón - tobón | dos**ú**f - d**ó**suf | dus**ú**f - dos**ú**f | duw**á**l - duw**á**l |
| 27 | 1 | /o:/-/u:/ | pof**á**t - pof**á**t | gob**á**l - g**ó**bal | gub**á**l - g**ó**bal | gus**í**f - gus**í**f |
| 28 | 1 | /o:/-/u:/ | botúl - botúl | kol**ó**p - k**ó**lop | kul**ó**p - kol**ó**p | kuf**á**t - kuf**á**t |
| 29 | 1 | /o:/-/u:/ | dot**á**p - dot**á**p | pot**á**n - p**ó**tan | put**á**n - pot**á**n | pus**í**m - pus**í**m |
| 30 | 1 | /o:/-/u:/ | kotám - kotám | tof**á**m - t**ó**fam | tuf**á**m - tof**á**m | tuk**á**p - tuk**á**p |
| 31 | 1 | /e:/-/ø:/ | pewál - pewál | bet**ú**l- b**é**tul | böt**ú**l - bet**ú**l | bör**á**f - bör**á**f |
| 32 | 1 | /e:/-/ø:/ | tekáp - tekáp | der**ó**m - d**é**rom | dör**ó**m - der**ó**m | döt**á**p - döt**á**p |
| 33 | 1 | /e:/-/ø:/ | kebút - kébut | ges**á**n - g**é**san | gös**á**n - ges**á**n | gös**í**f - gös**í**f |
| 34 | 1 | /e:/-/ø:/ | besíl - besíl | kel**ó**p - k**é**lop | köl**ó**p - kel**ó**p | köb**ú**f - köb**ú**f |
| 35 | 1 | /e:/-/ø:/ | demíl - demíl | peg**ú**s - p**é**gus | pög**ú**s - peg**ú**s | pös**í**m - pös**í**m |
| 36 | 1 | /e:/-/ø:/ | gebál - gebál | tef**á**m - t**é**fam | töf**á**m - t**é**fam | töb**á**r - töb**á**r |
| 37 | 1 | /u:/-/o:/ | gutúm - gutúm | bug**ó**m - b**ú**gom | bog**ó**m - bug**ó**m | bos**í**l - bos**í**l |
| 38 | 1 | /u:/-/o:/ | pugús - pugús | duw**á**l - d**ú**wal | dow**á**l - duw**á**l | dos**ú**f - dos**ú**f |
| 39 | 1 | /u:/-/o:/ | dolán - dolán | gus**í**f - g**ú**sif | gos**í**f - gus**í**f | gob**á**l - gob**á**l |
| 40 | 1 | /u:/-/o:/ | tufám - tufám | kuf**á**t - k**ú**fat | kof**á**t - kuf**á**t | kol**ó**p - kol**ó**p |
| 41 | 1 | /u:/-/o:/ | kubúf - kubúf | pus**í**m - p**ú**sim | pos**í**m - pus**í**m | pot**á**n - pot**á**n |
| 42 | 1 | /u:/-/o:/ | buráf - buráf | tuk**á**p - t**ú**kap | tok**á**p - tuk**á**p | tof**á**m - tof**á**m |
| 43 | 1 | /ø:/-/e:/ | töbón - töbón | bör**á**f - b**ö**raf | ber**á**f - bör**á**f | bet**ú**l - bet**ú**l |
| 44 | 1 | /ø:/-/e:/ | göt**ú**m - göt**ú**m | döt**á**p - d**ö**tap | det**á**p - döt**á**p | der**ó**m - der**ó**m |
| 45 | 1 | /ø:/-/e:/ | pötán - pötán | gös**í**f - g**ö**sif | ges**í**f - gös**í**f | ges**á**n - ges**á**n |
| 46 | 1 | /ø:/-/e:/ | dög**í**s - dög**í**s | köb**ú**f - k**ö**buf | keb**ú**f - köb**ú**f | kel**ó**p - kel**ó**p |
| 47 | 1 | /ø:/-/e:/ | kötám - kötám | pös**í**m - p**ö**sim | pes**í**m - pös**í**m | peg**ú**s - peg**ú**s |
| 48 | 1 | /ø:/-/e:/ | bögóm - bögóm | töb**á**r - t**ö**bar | teb**á**r - töb**á**r | tef**á**m - t**é**fam |
| 49 | 2 | /ø:/-/u:/ | púwal - pöwal | túrol - túrol | b**ö**sat - b**ö**sat | pöwal - pöwál |
| 50 | 2 | /ø:/-/u:/ | búnik - bönik | búwot - búwot | d**ö**mil - d**ö**mil | bönik - böník |
| 51 | 2 | /ø:/-/u:/ | dúrom - dörom | kúwit - kúwit | g**ö**san - g**ö**san | dörom - döróm |
| 52 | 2 | /ø:/-/u:/ | k**ú**sol - k**ö**sol | gúdam - gúdam | k**ö**but - k**ö**but | k**ö**sol - kös**ó**l |
| 53 | 2 | /ø:/-/u:/ | gúfol - göfol | púfat - púfat | p**ö**luk - p**ö**luk | göfol - göfól |
| 54 | 2 | /ø:/-/u:/ | túlum - tölum | dúsuf - dúsuf | t**ö**wök - t**ö**wök | t**ö**lum - töl**ú**m |
| 55 | 2 | /e:/-/o:/ | póluk - péluk | g**ó**nim - g**ó**nim | b**é**sat - b**é**sat | péluk - pelúk |
| 56 | 2 | /e:/-/o:/ | tólum - télum | póraf - póraf | d**é**gis - d**é**gis | télum - telúm |
| 57 | 2 | /e:/-/o:/ | kósol - késol | dólan - dólan | g**é**dam - g**é**dam | késol - kesól |
| 58 | 2 | /e:/-/o:/ | bónik - bénik | t**ó**wök - t**ó**wök | k**é**tam - k**é**tam | bénik - beník |
| 59 | 2 | /e:/-/o:/ | dósuf - désuf | kólat - kólat | p**é**fat - p**é**fat | désuf - desúf |
| 60 | 2 | /e:/-/o:/ | gófol - géfol | bówot - bówot | t**é**bon - t**é**bon | géfol - gefól |
| 61 | 2 | /u:/-/ø:/ | törol - túrol | pöwal - pöwal | b**ú**kap - b**ú**kap | túrol -turól |
| 62 | 2 | /u:/-/ø:/ | böwot - búwot | bönik - bönik | d**ú**ral - d**ú**ral | búwot - buwót |
| 63 | 2 | /u:/-/ø:/ | köwit - kúwit | dörom - dörom | g**ú**nim - g**ú**nim | kúwit - kuwít |
| 64 | 2 | /u:/-/ø:/ | gödam - gúdam | k**ö**sol - k**ö**sol | k**ú**tam - k**ú**tam | gúdam - gudám |
| 65 | 2 | /u:/-/ø:/ | pöfat - púfat | göfol - göfol | p**ú**raf - p**ú**raf | púfat - pufát |
| 66 | 2 | /u:/-/ø:/ | dösuf - dúsuf | t**ö**lum - t**ö**lum | t**ú**sit - t**ú**sit | dúsuf - dusúf |
| 67 | 2 | /o:/-/e:/ | g**é**nim - g**ó**nim | péluk - péluk | b**ó**kap - b**ó**kap | g**ó**nim - gon**í**m |
| 68 | 2 | /o:/-/e:/ | péraf - póraf | télum - télum | d**ó**wal - d**ó**wal | póraf - poráf |
| 69 | 2 | /o:/-/e:/ | délan - dólan | késol - késol | g**ó**sam - g**ó**sam | dólan - dolán |
| 70 | 2 | /o:/-/e:/ | t**é**wök - t**ó**wök | bénik - bénik | k**ó**fat - k**ó**fat | t**ó**wök - tow**ö**k |
| 71 | 2 | /o:/-/e:/ | kélat - kólat | désuf - désuf | p**ó**gus - p**ó**gus | kólat - kolát |
| 72 | 2 | /o:/-/e:/ | béwot - bówot | géfol - géfol | t**ó**sit - t**ó**sit | bówot - bowót |
| 73 | 2 | /ø:/-/u:/ | pöwál - pöwál | bös**á**t - b**ö**sat | bus**á**t - bös**á**t | buk**á**p - buk**á**p |
| 74 | 2 | /ø:/-/u:/ | böník - böník | döm**í**l - d**ö**mil | dum**í**l - döm**í**l | dur**á**l - dur**á**l |
| 75 | 2 | /ø:/-/u:/ | döróm - döróm | gös**á**n - g**ö**san | gus**á**n - gös**á**n | gun**í**m - gun**í**m |
| 76 | 2 | /ø:/-/u:/ | kös**ó**l - kös**ó**l | köb**ú**t - k**ö**but | kub**ú**t - köb**ú**t | kut**á**m - kut**á**m |
| 77 | 2 | /ø:/-/u:/ | göfól - göfól | pöl**ú**k - p**ö**luk | pul**ú**k - pöl**ú**k | pur**á**f - pur**á**f |
| 78 | 2 | /ø:/-/u:/ | töl**ú**m - töl**ú**m | töw**ö**k - t**ö**wök | tuw**ö**k - töw**ö**k | tus**í**t - tus**í**t |
| 79 | 2 | /e:/-/o:/ | pelúk - pelúk | bes**á**t - b**é**sat | b**o**s**á**t - bes**á**t | bok**á**p - bok**á**p |
| 80 | 2 | /e:/-/o:/ | telúm - telúm | deg**í**s - d**é**gis | dog**í**s - deg**í**s | dow**á**l - dow**á**l |
| 81 | 2 | /e:/-/o:/ | kesól - kesól | ged**á**m - g**é**dam | god**á**m - ged**á**m | gos**á**m - gos**á**m |
| 82 | 2 | /e:/-/o:/ | beník - beník | ket**á**m - k**é**tam | kot**á**m - ket**á**m | kof**á**t - kof**á**t |
| 83 | 2 | /e:/-/o:/ | desúf - desúf | pef**á**t - p**é**fat | pof**á**t - pef**á**t | pog**ú**s - pog**ú**s |
| 84 | 2 | /e:/-/o:/ | géfol - géfol | teb**ó**n - t**é**b**o**n | tob**ó**n - teb**ó**n | tos**í**t - tos**í**t |
| 85 | 2 | /u:/-/ø:/ | turól - turól | buk**á**p - b**ú**kap | bök**á**p - buk**á**p | bös**á**t - bös**á**t |
| 86 | 2 | /u:/-/ø:/ | buwót - buwót | dur**á**l - d**ú**ral | dör**á**l - dur**á**l | döm**í**l - döm**í**l |
| 87 | 2 | /u:/-/ø:/ | kuwít - kuwít | gun**í**m - g**ú**nim | gön**í**m - gun**í**m | gös**á**n - gös**á**n |
| 88 | 2 | /u:/-/ø:/ | gudám - gudám | kut**á**m - k**ú**tam | köt**á**m - kut**á**m | köb**ú**t - köb**ú**t |
| 89 | 2 | /u:/-/ø:/ | pufát - pufát | pur**á**f - p**ú**raf | pör**á**f - pur**á**f | pöl**ú**k - pöl**ú**k |
| 90 | 2 | /u:/-/ø:/ | dusúf - dusúf | tus**í**t - t**ú**sit | tös**í**t - tus**í**t | töw**ö**k - töw**ö**k |
| 91 | 2 | /o:/-/e:/ | gon**í**m - gon**í**m | bok**á**p - b**ó**kap | bek**á**p - bok**á**p | bes**á**t - bes**á**t |
| 92 | 2 | /o:/-/e:/ | poráf - poráf | dow**á**l - d**ó**wal | dew**á**l - dow**á**l | deg**í**s - deg**í**s |
| 93 | 2 | /o:/-/e:/ | dolán - dolán | gos**á**m - g**ó**sam | ges**á**m - gos**á**m | ged**á**m - ged**á**m |
| 94 | 2 | /o:/-/e:/ | tow**ö**k - tow**ö**k | kof**á**t - k**ó**fat | kef**á**t - kof**á**t | ket**á**m - ket**á**m |
| 95 | 2 | /o:/-/e:/ | kolát - kolát | pog**ú**s - p**ó**gus | peg**ú**s - pog**ú**s | pef**á**t - pef**á**t |
| 96 | 2 | /o:/-/e:/ | bowót - bowót | tos**í**t - t**ó**sit | tes**í**t - tos**í**t | teb**ó**n - teb**ó**n |
| 97 | 3 | /e:/-/u:/ | púraf - péraf | gúbal - gúbal | b**é**lot - b**é**lot | péraf -peráf |
| 98 | 3 | /e:/-/u:/ | kúbuf - kébuf | púluk - púluk | d**é**wal - d**é**wal | kébuf - kebúf |
| 99 | 3 | /e:/-/u:/ | dúlan - délan | d**ú**mil - d**ú**mil | g**é**tum - g**é**tum | délan - delán |
| 100 | 3 | /e:/-/u:/ | g**ú**sif - g**é**sif | túlum - túlum | k**é**mif - k**é**mif | g**é**sif - ges**í**f |
| 101 | 3 | /e:/-/u:/ | túsit - tésit | k**ú**sol - k**ú**sol | p**é**gis - p**é**gis | tésit - tesít |
| 102 | 3 | /e:/-/u:/ | búgom - bégom | bútul - bútul | t**é**bar - t**é**bar | bégom - begóm |
| 103 | 3 | /e:/-/u:/ | b**ú**raf - b**é**raf | gúfol - gúfol | b**é**kap - b**é**kap | b**é**raf - ber**á**f |
| 104 | 3 | /e:/-/u:/ | dútap - détap | púwal - púwal | d**é**ral - d**é**ral | détap - detáp |
| 105 | 3 | /e:/-/u:/ | t**ú**rol - t**é**rol | dúrom - dúrom | g**é**tol - g**é**tol | t**é**rol - ter**ó**l |
| 106 | 3 | /e:/-/u:/ | gúnim - génim | túkap - túkap | k**é**fat - k**é**fat | génim - gením |
| 107 | 3 | /e:/-/u:/ | k**ú**wit - k**é**wit | kúlap - kúlap | p**é**sal - p**é**sal | k**é**wit - kew**í**t |
| 108 | 3 | /e:/-/u:/ | púsim - pésim | búsil - búsil | t**é**map - t**é**map | pésim - pesím |
| 109 | 3 | /u:/-/e:/ | gébal - gúbal | b**é**raf - b**é**raf | b**ú**nik - b**ú**nik | gúbal - gubál |
| 110 | 3 | /u:/-/e:/ | péluk - púluk | détap - détap | d**ú**sim - d**ú**sim | púluk - pulúk |
| 111 | 3 | /u:/-/e:/ | d**é**mil - d**ú**mil | t**é**rol - t**é**rol | g**ú**lan - g**ú**lan | d**ú**mil - dum**í**l |
| 112 | 3 | /u:/-/e:/ | télum - túlum | génim - génim | k**ú**but - k**ú**but | túlum - tulúm |
| 113 | 3 | /u:/-/e:/ | k**é**sol - k**ú**sol | k**é**wit - k**é**wit | p**ú**tul - p**ú**tul | k**ú**sol - kus**ó**l |
| 114 | 3 | /u:/-/e:/ | bétul - bútul | pésim - pésim | t**ú**mil - t**ú**mil | bútul - butúl |
| 115 | 3 | /u:/-/e:/ | géfol - gúfol | péraf - péraf | b**ú**sat - b**ú**sat | gúfol - gufól |
| 116 | 3 | /u:/-/e:/ | péwal - púwal | kébuf - kébuf | d**ú**mik - d**ú**mik | púwal - puwál |
| 117 | 3 | /u:/-/e:/ | dérom - dúrom | délan - délan | g**ú**but - g**ú**but | dúrom - duróm |
| 118 | 3 | /u:/-/e:/ | tékap - túkap | g**é**sif - g**é**sif | k**ú**raf - k**ú**raf | túkap - tukáp |
| 119 | 3 | /u:/-/e:/ | kélap - kúlap | tésit - tésit | p**ú**rol - p**ú**rol | kúlap - kuláp |
| 120 | 3 | /u:/-/e:/ | bésil - búsil | bégom - bégom | t**ú**gis - t**ú**gis | búsil - busíl |
| 121 | 3 | /e:/-/u:/ | peráf - peráf | bel**ó**t - b**é**lot | bul**ó**t - bel**ó**t | bek**á**p - bek**á**p |
| 122 | 3 | /e:/-/u:/ | kebúf - kebúf | dew**á**l - d**é**wal | duw**á**l - dew**á**l | der**á**l - der**á**l |
| 123 | 3 | /e:/-/u:/ | delán - delán | get**ú**m - g**é**tum | gut**ú**m - get**ú**m | get**ó**l - get**ó**l |
| 124 | 3 | /e:/-/u:/ | ges**í**f - ges**í**f | kem**í**f - k**é**mif | kum**í**f - kem**í**f | kef**á**t - kef**á**t |
| 125 | 3 | /e:/-/u:/ | tesít - tesít | peg**í**s - p**é**gis | pug**í**s - peg**í**s | pes**á**l - pes**á**l |
| 126 | 3 | /e:/-/u:/ | begóm - begóm | teb**á**r - t**é**bar | tub**á**r - teb**á**r | tem**á**p - tem**á**p |
| 127 | 3 | /e:/-/u:/ | ber**á**f - ber**á**f | bek**á**p - b**é**kap | buk**á**p - bek**á**p | bel**ó**t - bel**ó**t |
| 128 | 3 | /e:/-/u:/ | detáp - detáp | der**á**l - d**é**ral | dur**á**l - der**á**l | dew**á**l - dew**á**l |
| 129 | 3 | /e:/-/u:/ | ter**ó**l - ter**ó**l | get**ó**l - g**é**tol | gut**ó**l - get**ó**l | get**ú**m - get**ú**m |
| 130 | 3 | /e:/-/u:/ | gením - gením | kef**á**t - k**é**fat | kuf**á**t - kef**á**t | kem**í**f - kem**í**f |
| 131 | 3 | /e:/-/u:/ | kew**í**t - kew**í**t | pes**á**l - p**é**sal | pus**á**l - pes**á**l | peg**í**s - peg**í**s |
| 132 | 3 | /e:/-/u:/ | pesím - pesím | tem**á**p - t**é**map | tum**á**p - tem**á**p | teb**á**r - teb**á**r |
| 133 | 3 | /u:/-/e:/ | gubál - gubál | bun**í**k - b**ú**nik | ben**í**k - bun**í**k | bus**á**t - bus**á**t |
| 134 | 3 | /u:/-/e:/ | pulúk - pulúk | dus**í**m - d**ú**sim | des**í**m - dus**í**m | dum**í**k - dum**í**k |
| 135 | 3 | /u:/-/e:/ | dum**í**l - dum**í**l | gul**á**n - g**ú**lan | gel**á**n - gul**á**n | gub**ú**t - gub**ú**t |
| 136 | 3 | /u:/-/e:/ | tulúm - tulúm | kub**ú**t - k**ú**but | keb**ú**t - kub**ú**t | kur**á**f - kur**á**f |
| 137 | 3 | /u:/-/e:/ | kus**ó**l - kus**ó**l | put**ú**l - p**ú**tul | pet**ú**l - put**ú**l | pur**ó**l - pur**ó**l |
| 138 | 3 | /u:/-/e:/ | butúl - butúl | tum**í**l - t**ú**mil | tem**í**l - tum**í**l | tug**í**s - tug**í**s |
| 139 | 3 | /u:/-/e:/ | gufól - gufól | bus**á**t - b**ú**sat | bes**á**t - bus**á**t | bun**í**k - bun**í**k |
| 140 | 3 | /u:/-/e:/ | puwál - puwál | dum**í**k - d**ú**mik | dem**í**k - dum**í**k | dus**í**m - dus**í**m |
| 141 | 3 | /u:/-/e:/ | duróm - duróm | gub**ú**t - g**ú**but | geb**ú**t - gub**ú**t | gul**á**n - gul**á**n |
| 142 | 3 | /u:/-/e:/ | tukáp - tukáp | kur**á**f - k**ú**raf | ker**á**f - kur**á**f | kub**ú**t - kub**ú**t |
| 143 | 3 | /u:/-/e:/ | kuláp - kuláp | pur**ó**l - p**ú**rol | per**ó**l - pur**ó**l | put**ú**l - put**ú**l |
| 144 | 3 | /u:/-/e:/ | busíl - busíl | tug**í**s - t**ú**gis | teg**í**s - tug**í**s | tum**í**l - tum**í**l |
|  |  |  |  |  |  |  |
|  | **Vowel quality** | | **Control pairs** | | | |
|  | **Feature** | | **Penultimate stress** | | **Final stress** | |
|  | **difference** | | **Vowel quality** | **Stress** | **Vowel quality** | **Stress** |
| 1 | 1 | /o:/-/u:/ |  | gódam - gódam | godám - godám |  |
| 2 | 1 | /o:/-/u:/ |  | tóbon - tóbon | tobón - tobón |  |
| 3 | 1 | /o:/-/u:/ |  | p**ó**fat - p**ó**fat | pof**á**t - pof**á**t |  |
| 4 | 1 | /o:/-/u:/ |  | bótul - bótul | botúl - botúl |  |
| 5 | 1 | /o:/-/u:/ |  | d**ó**tap - d**ó**tap | dot**á**p - dot**á**p |  |
| 6 | 1 | /o:/-/u:/ |  | kótam - kótam | kotám - kotám |  |
| 7 | 1 | /e:/-/ø:/ |  | péwal - péwal | pewál - pewál |  |
| 8 | 1 | /e:/-/ø:/ |  | tékap - tékap | tekáp - tekáp |  |
| 9 | 1 | /e:/-/ø:/ |  | kébut - kébut | kebút - kébut |  |
| 10 | 1 | /e:/-/ø:/ |  | bésil - bésil | besíl - besíl |  |
| 11 | 1 | /e:/-/ø:/ |  | démil - démil | demíl - demíl |  |
| 12 | 1 | /e:/-/ø:/ |  | gébal - gébal | gebál - gebál |  |
| 13 | 1 | /u:/-/o:/ |  | gútum - gútum | gutúm - gutúm |  |
| 14 | 1 | /u:/-/o:/ |  | púgus - púgus | pugús - pugús |  |
| 15 | 1 | /u:/-/o:/ |  | dúlan - dúlan | dulán - dulán |  |
| 16 | 1 | /u:/-/o:/ |  | túfam - túfam | tufám - tufám |  |
| 17 | 1 | /u:/-/o:/ |  | kúbuf - kúbuf | kubúf - kubúf |  |
| 18 | 1 | /u:/-/o:/ |  | búraf - búraf | buráf - buráf |  |
| 19 | 1 | /ø:/-/e:/ |  | töbon - töbon | töbón - töbón |  |
| 20 | 1 | /ø:/-/e:/ |  | g**ö**tum - g**ö**tum | göt**ú**m - göt**ú**m |  |
| 21 | 1 | /ø:/-/e:/ |  | pötan - pötan | pötán - pötán |  |
| 22 | 1 | /ø:/-/e:/ |  | d**ö**gis - d**ö**gis | dög**í**s - dög**í**s |  |
| 23 | 1 | /ø:/-/e:/ |  | kötam - kötam | kötám - kötám |  |
| 24 | 1 | /ø:/-/e:/ |  | bögom - bögom | bögóm - bögóm |  |
| 25 | 1 | /o:/-/u:/ | b**ó**sil - b**ó**sil |  |  | bos**í**l - bos**í**l |
| 26 | 1 | /o:/-/u:/ | d**ó**suf - d**ó**s**u**f |  |  | dos**ú**f - dos**ú**f |
| 27 | 1 | /o:/-/u:/ | g**ó**bal - g**ó**bal |  |  | gob**á**l - gob**á**l |
| 28 | 1 | /o:/-/u:/ | k**ó**lop - k**ó**lop |  |  | kol**ó**p - kol**ó**p |
| 29 | 1 | /o:/-/u:/ | p**ó**tan - p**ó**t**a**n |  |  | pot**á**n - pot**á**n |
| 30 | 1 | /o:/-/u:/ | t**ó**fam - t**ó**fam |  |  | tof**á**m - tof**á**m |
| 31 | 1 | /e:/-/ø:/ | b**é**tul - b**é**tul |  |  | bet**ú**l - bet**ú**l |
| 32 | 1 | /e:/-/ø:/ | d**é**rom - d**é**rom |  |  | der**ó**m - der**ó**m |
| 33 | 1 | /e:/-/ø:/ | g**é**san - g**é**san |  |  | ges**á**n - ges**á**n |
| 34 | 1 | /e:/-/ø:/ | k**é**lop - k**é**lop |  |  | kel**ó**p - kel**ó**p |
| 35 | 1 | /e:/-/ø:/ | p**é**gus - p**é**gus |  |  | peg**ú**s - peg**ú**s |
| 36 | 1 | /e:/-/ø:/ | t**é**fam - t**é**fam |  |  | tef**á**m - t**é**fam |
| 37 | 1 | /u:/-/o:/ | b**ú**gom - b**ú**gom |  |  | bug**ó**m - bug**ó**m |
| 38 | 1 | /u:/-/o:/ | d**ú**wal - d**ú**wal |  |  | duw**á**l - duw**á**l |
| 39 | 1 | /u:/-/o:/ | g**ú**sif - g**ú**sif |  |  | gus**í**f - gus**í**f |
| 40 | 1 | /u:/-/o:/ | k**ú**fat - k**ú**fat |  |  | kuf**á**t - kuf**á**t |
| 41 | 1 | /u:/-/o:/ | p**ú**sim - p**ú**sim |  |  | pus**í**m - pus**í**m |
| 42 | 1 | /u:/-/o:/ | t**ú**kap - t**ú**kap |  |  | tuk**á**p - tuk**á**p |
| 43 | 1 | /ø:/-/e:/ | b**ö**raf - b**ö**raf |  |  | bör**á**f - bör**á**f |
| 44 | 1 | /ø:/-/e:/ | d**ö**tap - d**ö**tap |  |  | döt**á**p - döt**á**p |
| 45 | 1 | /ø:/-/e:/ | g**ö**sif - g**ö**sif |  |  | gös**í**f - gös**í**f |
| 46 | 1 | /ø:/-/e:/ | k**ö**buf - k**ö**buf |  |  | köb**ú**f - köb**ú**f |
| 47 | 1 | /ø:/-/e:/ | p**ö**sim - p**ö**sim |  |  | pös**í**m - pös**í**m |
| 48 | 1 | /ø:/-/e:/ | t**ö**bar - t**ö**bar |  |  | töb**á**r - töb**á**r |
| 49 | 2 | /ø:/-/u:/ |  | pöwal - pöwal | pöwál - pöwál |  |
| 50 | 2 | /ø:/-/u:/ |  | bönik - bönik | böník - böník |  |
| 51 | 2 | /ø:/-/u:/ |  | dörom - dörom | döróm - döróm |  |
| 52 | 2 | /ø:/-/u:/ |  | k**ö**sol - k**ö**sol | kös**ó**l - kös**ó**l |  |
| 53 | 2 | /ø:/-/u:/ |  | göfol - göfol | göfól - göfól |  |
| 54 | 2 | /ø:/-/u:/ |  | t**ö**lum - t**ö**lum | töl**ú**m - töl**ú**m |  |
| 55 | 2 | /e:/-/o:/ |  | péluk - péluk | pelúk - pelúk |  |
| 56 | 2 | /e:/-/o:/ |  | télum - télum | telúm - telúm |  |
| 57 | 2 | /e:/-/o:/ |  | késol - késol | kesól - kesól |  |
| 58 | 2 | /e:/-/o:/ |  | bénik - bénik | beník - beník |  |
| 59 | 2 | /e:/-/o:/ |  | désuf - désuf | desúf - desúf |  |
| 60 | 2 | /e:/-/o:/ |  | géfol - géfol | géfol - géfol |  |
| 61 | 2 | /u:/-/ø:/ |  | túrol - túrol | turól - turól |  |
| 62 | 2 | /u:/-/ø:/ |  | búwot - búwot | buwót - buwót |  |
| 63 | 2 | /u:/-/ø:/ |  | kúwit - kúwit | kuwít - kuwít |  |
| 64 | 2 | /u:/-/ø:/ |  | gúdam - gúdam | gudám - gudám |  |
| 65 | 2 | /u:/-/ø:/ |  | púfat - púfat | pufát - pufát |  |
| 66 | 2 | /u:/-/ø:/ |  | dúsuf - dúsuf | dusúf - dusúf |  |
| 67 | 2 | /o:/-/e:/ |  | g**ó**nim - g**ó**nim | gon**í**m - gon**í**m |  |
| 68 | 2 | /o:/-/e:/ |  | póraf - póraf | poráf - poráf |  |
| 69 | 2 | /o:/-/e:/ |  | dólan - dólan | dolán - dolán |  |
| 70 | 2 | /o:/-/e:/ |  | t**ó**wök - t**ó**wök | tow**ö**k - tow**ö**k |  |
| 71 | 2 | /o:/-/e:/ |  | kólat - kólat | kolát - kolát |  |
| 72 | 2 | /o:/-/e:/ |  | bówot - bówot | bowót - bowót |  |
| 73 | 2 | /ø:/-/u:/ | b**ö**sat - b**ö**sat |  |  | bös**á**t - bös**á**t |
| 74 | 2 | /ø:/-/u:/ | d**ö**mil - d**ö**mil |  |  | döm**í**l - döm**í**l |
| 75 | 2 | /ø:/-/u:/ | g**ö**san - g**ö**san |  |  | gös**á**n - gös**á**n |
| 76 | 2 | /ø:/-/u:/ | k**ö**but - k**ö**but |  |  | köb**ú**t - köb**ú**t |
| 77 | 2 | /ø:/-/u:/ | p**ö**luk - p**ö**luk |  |  | pöl**ú**k - pöl**ú**k |
| 78 | 2 | /ø:/-/u:/ | t**ö**wök - t**ö**wök |  |  | töw**ö**k - töw**ö**k |
| 79 | 2 | /e:/-/o:/ | b**é**sat - b**é**sat |  |  | bes**á**t - bes**á**t |
| 80 | 2 | /e:/-/o:/ | d**é**gis - d**é**gis |  |  | deg**í**s - deg**í**s |
| 81 | 2 | /e:/-/o:/ | g**é**dam - g**é**dam |  |  | ged**á**m - ged**á**m |
| 82 | 2 | /e:/-/o:/ | k**é**tam - k**é**tam |  |  | ket**á**m - ket**á**m |
| 83 | 2 | /e:/-/o:/ | p**é**fat - p**é**fat |  |  | pef**á**t - pef**á**t |
| 84 | 2 | /e:/-/o:/ | t**é**bon - t**é**bon |  |  | teb**ó**n - teb**ó**n |
| 85 | 2 | /u:/-/ø:/ | b**ú**kap - b**ú**kap |  |  | buk**á**p - buk**á**p |
| 86 | 2 | /u:/-/ø:/ | d**ú**ral - d**ú**ral |  |  | dur**á**l - dur**á**l |
| 87 | 2 | /u:/-/ø:/ | g**ú**nim - g**ú**nim |  |  | gun**í**m - gun**í**m |
| 88 | 2 | /u:/-/ø:/ | k**ú**tam - k**ú**tam |  |  | kut**á**m - kut**á**m |
| 89 | 2 | /u:/-/ø:/ | p**ú**raf - p**ú**raf |  |  | pur**á**f - pur**á**f |
| 90 | 2 | /u:/-/ø:/ | t**ú**sit - t**ú**sit |  |  | tus**í**t - tus**í**t |
| 91 | 2 | /o:/-/e:/ | b**ó**kap - b**ó**kap |  |  | bok**á**p - bok**á**p |
| 92 | 2 | /o:/-/e:/ | d**ó**wal - d**ó**wal |  |  | dow**á**l - dow**á**l |
| 93 | 2 | /o:/-/e:/ | g**ó**sam - g**ó**sam |  |  | gos**á**m - gos**á**m |
| 94 | 2 | /o:/-/e:/ | k**ó**fat - k**ó**fat |  |  | kof**á**t - kof**á**t |
| 95 | 2 | /o:/-/e:/ | p**ó**gus - p**ó**gus |  |  | pog**ú**s - pog**ú**s |
| 96 | 2 | /o:/-/e:/ | t**ó**sit - t**ó**sit |  |  | tos**í**t - tos**í**t |
| 97 | 3 | /e:/-/u:/ |  | péraf - péraf | peráf - peráf |  |
| 98 | 3 | /e:/-/u:/ |  | kébuf - kébuf | kebúf - kebúf |  |
| 99 | 3 | /e:/-/u:/ |  | délan - délan | delán - delán |  |
| 100 | 3 | /e:/-/u:/ |  | g**é**sif - g**é**sif | ges**í**f - ges**í**f |  |
| 101 | 3 | /e:/-/u:/ |  | tésit - tésit | tesít - tesít |  |
| 102 | 3 | /e:/-/u:/ |  | bégom - bégom | begóm - begóm |  |
| 103 | 3 | /e:/-/u:/ |  | b**é**raf - b**é**raf | ber**á**f - ber**á**f |  |
| 104 | 3 | /e:/-/u:/ |  | détap - détap | detáp - detáp |  |
| 105 | 3 | /e:/-/u:/ |  | t**é**rol - t**é**rol | ter**ó**l - ter**ó**l |  |
| 106 | 3 | /e:/-/u:/ |  | génim - génim | gením - gením |  |
| 107 | 3 | /e:/-/u:/ |  | k**é**wit - k**é**wit | kew**í**t - kew**í**t |  |
| 108 | 3 | /e:/-/u:/ |  | pésim - pésim | pesím - pesím |  |
| 109 | 3 | /u:/-/e:/ |  | gúbal - gúbal | gubál - gubál |  |
| 110 | 3 | /u:/-/e:/ |  | púluk - púluk | pulúk - pulúk |  |
| 111 | 3 | /u:/-/e:/ |  | d**ú**mil - d**ú**mil | dum**í**l - dum**í**l |  |
| 112 | 3 | /u:/-/e:/ |  | túlum - túlum | tulúm - tulúm |  |
| 113 | 3 | /u:/-/e:/ |  | k**ú**sol - k**ú**sol | kus**ó**l - kus**ó**l |  |
| 114 | 3 | /u:/-/e:/ |  | bútul - bútul | butúl - butúl |  |
| 115 | 3 | /u:/-/e:/ |  | gúfol - gúfol | gufól - gufól |  |
| 116 | 3 | /u:/-/e:/ |  | púwal - púwal | puwál - puwál |  |
| 117 | 3 | /u:/-/e:/ |  | dúrom - dúrom | duróm - duróm |  |
| 118 | 3 | /u:/-/e:/ |  | túkap - túkap | tukáp - tukáp |  |
| 119 | 3 | /u:/-/e:/ |  | kúlap - kúlap | kuláp - kuláp |  |
| 120 | 3 | /u:/-/e:/ |  | búsil - búsil | busíl - busíl |  |
| 121 | 3 | /e:/-/u:/ | b**é**lot - b**é**lot |  |  | bel**ó**t - bel**ó**t |
| 122 | 3 | /e:/-/u:/ | d**é**wal - d**é**wal |  |  | dew**á**l - dew**á**l |
| 123 | 3 | /e:/-/u:/ | g**é**tum - g**é**tum |  |  | get**ú**m - get**ú**m |
| 124 | 3 | /e:/-/u:/ | k**é**mif - k**é**mif |  |  | kem**í**f - kem**í**f |
| 125 | 3 | /e:/-/u:/ | p**é**gis - p**é**gis |  |  | peg**í**s - peg**í**s |
| 126 | 3 | /e:/-/u:/ | t**é**bar - t**é**bar |  |  | teb**á**r - teb**á**r |
| 127 | 3 | /e:/-/u:/ | b**é**kap - b**é**kap |  |  | bek**á**p - bek**á**p |
| 128 | 3 | /e:/-/u:/ | d**é**ral - d**é**ral |  |  | der**á**l - der**á**l |
| 129 | 3 | /e:/-/u:/ | g**é**tol - g**é**tol |  |  | get**ó**l - get**ó**l |
| 130 | 3 | /e:/-/u:/ | k**é**fat - k**é**fat |  |  | kef**á**t - kef**á**t |
| 131 | 3 | /e:/-/u:/ | p**é**sal - p**é**sal |  |  | pes**á**l - pes**á**l |
| 132 | 3 | /e:/-/u:/ | t**é**map - t**é**map |  |  | tem**á**p - tem**á**p |
| 133 | 3 | /u:/-/e:/ | b**ú**nik - b**ú**nik |  |  | bun**í**k - bun**í**k |
| 134 | 3 | /u:/-/e:/ | d**ú**sim - d**ú**sim |  |  | dus**í**m - dus**í**m |
| 135 | 3 | /u:/-/e:/ | g**ú**lan - g**ú**lan |  |  | gul**á**n - gul**á**n |
| 136 | 3 | /u:/-/e:/ | k**ú**but - k**ú**but |  |  | kub**ú**t - kub**ú**t |
| 137 | 3 | /u:/-/e:/ | p**ú**tul - p**ú**tul |  |  | put**ú**l - put**ú**l |
| 138 | 3 | /u:/-/e:/ | t**ú**mil - t**ú**mil |  |  | tum**í**l - tum**í**l |
| 139 | 3 | /u:/-/e:/ | b**ú**sat - b**ú**sat |  |  | bus**á**t - bus**á**t |
| 140 | 3 | /u:/-/e:/ | d**ú**mik - d**ú**mik |  |  | dum**í**k - dum**í**k |
| 141 | 3 | /u:/-/e:/ | g**ú**but - g**ú**but |  |  | gub**ú**t - gub**ú**t |
| 142 | 3 | /u:/-/e:/ | k**ú**raf - k**ú**raf |  |  | kur**á**f - kur**á**f |
| 143 | 3 | /u:/-/e:/ | p**ú**rol - p**ú**rol |  |  | pur**ó**l - pur**ó**l |
| 144 | 3 | /u:/-/e:/ | t**ú**gis - t**ú**gis |  |  | tug**í**s - tug**í**s |
